# Experience in the Management of Peripancreatic Collections Using Endoscopic Ultrasound in a Tertiary Referral Hospital in Mexico

**DOI:** 10.7759/cureus.113183

**Published:** 2026-07-22

**Authors:** Diomedes de Jesús Durango Hernández, Yoeli Marisa Escandon Espinoza, Katia Daniela Lopez Garcia

**Affiliations:** 1 Gastrointestinal Endoscopy/General Surgery, Hospital Regional de Alta Especialidad Bicentenario de la Independencia, Instituto de Seguridad y Servicios Sociales de los Trabajadores del Estado (ISSSTE), Tultitlan, MEX; 2 Gastrointestinal Endoscopy, Hospital Regional de Alta Especialidad Bicentenario de la Independencia, Instituto de Seguridad y Servicios Sociales de los Trabajadores del Estado (ISSSTE), Tultitlan, MEX

**Keywords:** endoscopic ultrasound, eus-guided drainage, pancreatic pseudocyst, peripancreatic fluid collections, walled-off necrosis

## Abstract

Introduction: Acute pancreatitis (AP) accounts for approximately 230,000 hospital admissions per year in the United States alone. Peripancreatic fluid collections (PFCs) are recognized complications of AP. Currently, the management of PFCs has evolved toward a minimally invasive step-up approach, in which endoscopic ultrasound (EUS)-guided drainage has progressively become the standard of care.

Methods: A retrospective observational study was conducted, including patients with peripancreatic collections who underwent EUS-guided drainage over a three-year period at the Hospital Regional de Alta Especialidad Bicentenario de la Independencia, Instituto de Seguridad y Servicios Sociales de los Trabajadores del Estado (ISSSTE), located in Tultitlán, State of Mexico, Mexico.

Results: A total of 35 patients underwent EUS-guided endoscopic drainage of peripancreatic collections. The mean age was 49.68 ± 12.90 years, and female patients were slightly predominant (51.4%). Pancreatic pseudocyst was the most frequent type of collection, observed in 65.7% of cases (n = 23), while walled-off necrosis (WON) accounted for 34.3% (n = 12). The most commonly used stent was the double pigtail plastic stent, employed in 60.0% of patients, compared with the lumen-apposing metal stent (LAMS), used in 40.0%. The mean stent indwelling time was 35.31 ± 9.58 days, with a mean post-procedure hospital stay of 3.5 days. Technical success was achieved in 100% of cases. Only three recurrences were observed (8.6%). Complications occurred in 5.8% of patients, and no 30-day mortality was reported (n = 0).

Conclusions: EUS-guided endoscopic drainage of peripancreatic collections demonstrated high efficacy and an acceptable safety profile.

## Introduction

Acute pancreatitis (AP) accounts for approximately 230,000 hospital admissions per year in the United States alone. A growing concern is that 20% of cases develop a severe form of the disease, associated with a mortality risk ranging from 10% to 30% [1]. Gallstone disease remains the most common cause of AP (40-70%), predominantly affecting women, while excessive alcohol consumption ranks second (25-30%) and is more commonly associated with AP in men [2].

Although most cases follow a mild and self-limited course, 10% to 20% progress to severe disease with systemic and local complications that worsen organ failure and increase the risk of mortality [3]. Peripancreatic fluid collections (PFCs) are recognized complications of AP and result from disruption of the pancreatic duct, leading to fluid accumulation in the retroperitoneal or peripancreatic spaces [4].

According to the revised Atlanta classification (2012), these collections are classified as acute peripancreatic fluid collections and pancreatic pseudocysts, the latter considered a late complication occurring after four weeks of evolution [4]. In addition, pancreatic necrosis (5-10%) may lead to acute necrotic collections or walled-off necrosis (WON), characterized by a well-defined radiological capsule that develops late (>4 weeks) [5]. Understanding the concept of a “wall” is essential from an endoscopic perspective, as it allows safe puncture into a confined and well-demarcated cavity [6].

Most pancreatic pseudocysts are asymptomatic and tend to resolve spontaneously; however, those persisting beyond six weeks or associated with clinical symptoms require drainage. Drainage may be performed via a transpapillary endoscopic approach in cases with communication to the pancreatic duct, or via transmural drainage when the pseudocyst is directly adjacent to the gastroduodenal wall [7].

Historically, patients with necrotic collections underwent laparotomy; however, this approach was associated with high complication and mortality rates. This paradigm has changed dramatically over the past 20 years. Following the publication of the PANTER trial in 2010, a step-up approach was proposed for the management of necrotic collections using minimally invasive techniques, which allow a progressive treatment strategy based on the patient’s clinical evolution [2,8]. This approach has demonstrated success rates of 97% to 99%. Similarly, the PENGUIN trial (2012) showed that endoscopic necrosectomy is associated with fewer complications, establishing the step-up approach as the current standard of care [9,10].

Current indications for drainage include symptomatic or infected pseudocysts or pancreatic necrosis [11]. It is also indicated in cases of extrinsic compression causing intestinal or biliary obstruction, as well as abdominal compartment syndrome. Early drainage (<2 weeks) should generally be avoided, and delayed drainage (>3-4 weeks) is preferred whenever clinically feasible [12].

Initially, endoscopic drainage was performed by “blind” puncture of the submucosal bulge caused by large collections, increasing the risk of complications such as bleeding and perforation. In recent years, endoscopic ultrasound (EUS)-guided drainage has been widely adopted and has progressively become the standard of care [7]. EUS plays a key role in the evaluation and management of PFCs and allows drainage using the main technique: a single-step technique using a linear EUS scope, allowing puncture through the working channel, guidewire placement, tract dilation with a balloon, and stent deployment [8].

Stents used for this procedure may be plastic or metal. Metal stents have shown better outcomes due to their larger luminal diameter and facilitation of endoscopic necrosectomy. Among them, lumen-apposing metal stents (LAMS) are designed to minimize the risk of migration [12]. Hot LAMS feature an electrocautery-enhanced delivery system. They allow the endoscopist to puncture and deploy the stent in one streamlined step directly through the target wall, which reduces procedural time and lowers the risk of losing access to the target area. Cold LAMS lack a built-in cautery tip. They require a multi-step approach: puncturing the target with a needle, placing a guidewire, dilating the tract with a balloon, and then manually passing the stent over the wire.

Despite high technical success rates of EUS-guided drainage, immediate or delayed complications may occur. These are often related to complex anatomical locations, such as collections in the uncinate process or those located more than 1 cm from the gastroduodenal wall, as well as stent migration, obstruction, bleeding, or perforation [6].

Nevertheless, EUS-guided drainage is associated with high technical and clinical success rates; therefore, transmural drainage is currently considered the standard of care for patients with peripancreatic collections [6].

## Materials and methods

A descriptive retrospective study was conducted, including 35 patients with peripancreatic collections as a complication of acute pancreatitis who underwent EUS-guided drainage between January 2023 and March 2026 at the Hospital Regional de Alta Especialidad Bicentenario de la Independencia, Instituto de Seguridad y Servicios Sociales de los Trabajadores del Estado (ISSSTE), located in Tultitlán, State of Mexico, Mexico.

Patients with local complications persisting for more than four weeks and ongoing symptoms were included. These were defined as pancreatic pseudocysts and WON, with or without evidence of infection.

Case identification was performed through review of the institutional Endoscopy Unit database, using diagnostic codes related to peripancreatic collections and EUS-guided drainage procedures. EUS-guided drainage was performed by experienced endoscopists using a linear-array echoendoscope. The target collection was identified and evaluated regarding size, contents, wall maturity, and proximity to adjacent structures. After an optimal access point was identified, the collection was punctured under EUS guidance using a 19-gauge needle. The transmural tract was subsequently dilated using a balloon dilator, according to the characteristics of the collection and the endoscopist’s preference.

Variables analyzed included age, sex, and type of pancreatic collection, classified as pancreatic pseudocyst or WON, with or without infection. Technical success was defined as complete resolution of the cavity and associated symptoms after the initial intervention. Recurrence was defined as the reappearance of the collection associated with clinical symptoms more than four weeks after stent removal.

The type of stent used was recorded, classified as a double pigtail plastic stent or a LAMS. Stent indwelling time (in days) and post-procedural hospital stay were also analyzed.

The transmural drainage route (gastric or duodenal) was documented, as well as procedure-related complications, including stent obstruction or migration, bleeding, and perforation. Technical failure was defined as the inability to perform a puncture due to the unfavorable anatomical location of the collection. Thirty-day mortality was also recorded.

Patients with cystic neoplasms misclassified as pancreatic collections, those without post-procedural follow-up, and those with incomplete medical records were excluded.

The statistical analysis was descriptive, so categorical variables were reported as percentages; non-normally distributed variables were described using medians and interquartile ranges, and normally distributed variables were described using mean and standard deviation (SD).

The study protocol was reviewed and approved by the Research and Ethics Committee of the Hospital Regional de Alta Especialidad Bicentenario de la Independencia, ISSSTE. A literature review on peripancreatic collections and their endoscopic management was conducted using national and international databases. Based on available evidence and review by specialists in general surgery, gastrointestinal endoscopy, and endoscopic ultrasound, the study conclusions were formulated.

## Results

EUS-guided endoscopic drainage of peripancreatic collections was performed in a total of 35 patients over a three-year period. The mean age was 49.68 ± 12.90 years, with a range from 28 to 86 years. There was a slight female predominance, accounting for 51.4% (n = 18), while males represented 48.6% (n = 17).

**Figure 1:**
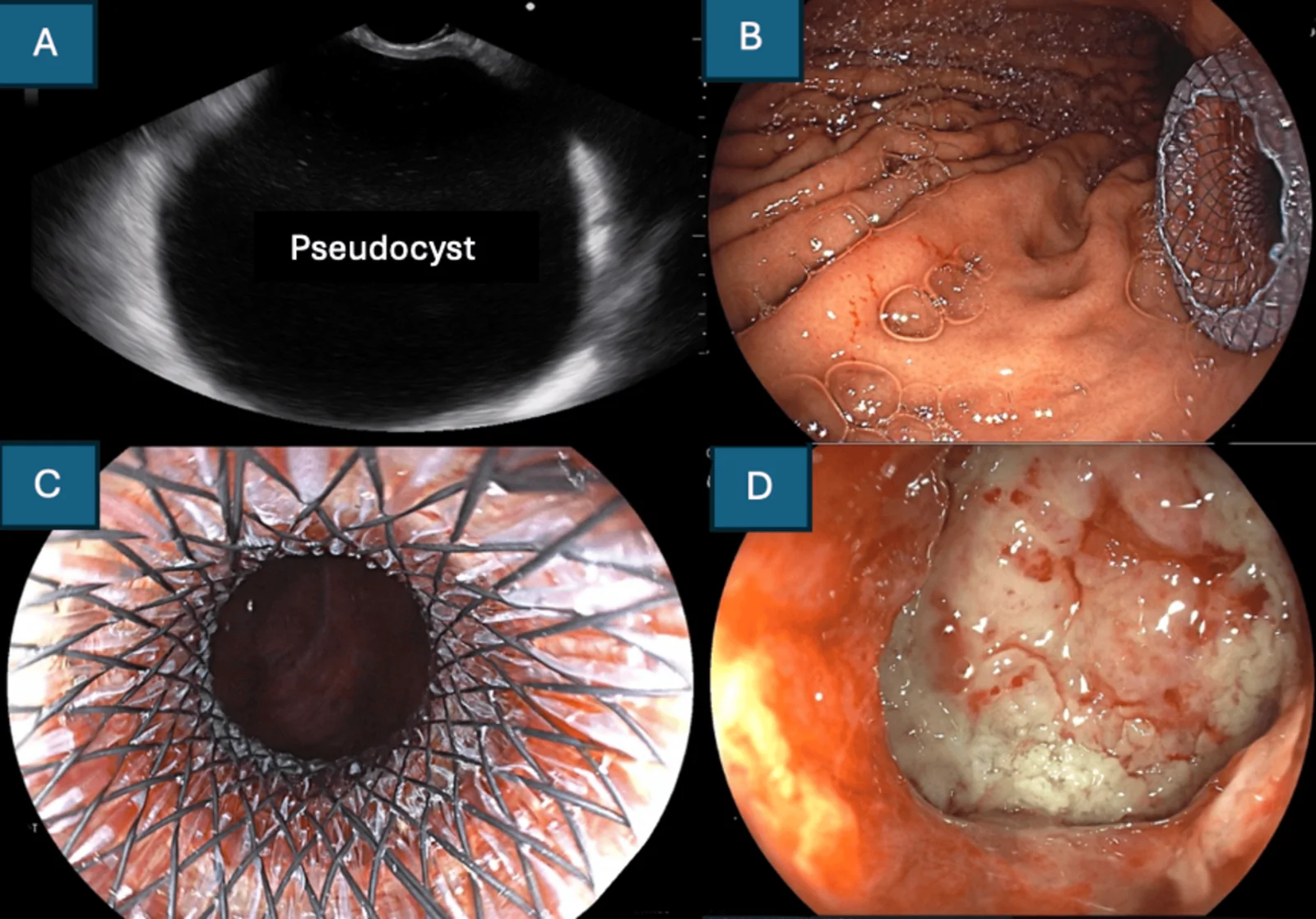
Endoscopic ultrasound (EUS) view of a 10-cm pancreatic pseudocyst. (A) EUS view of a 10-cm pancreatic pseudocyst. (B) Drainage using a lumen-apposing metal stent (LAMS). (C) Endoscopic view of the interior of the cystic cavity. (D) Resolution of the cystic cavity four weeks after drainage.

**Figure 2:**
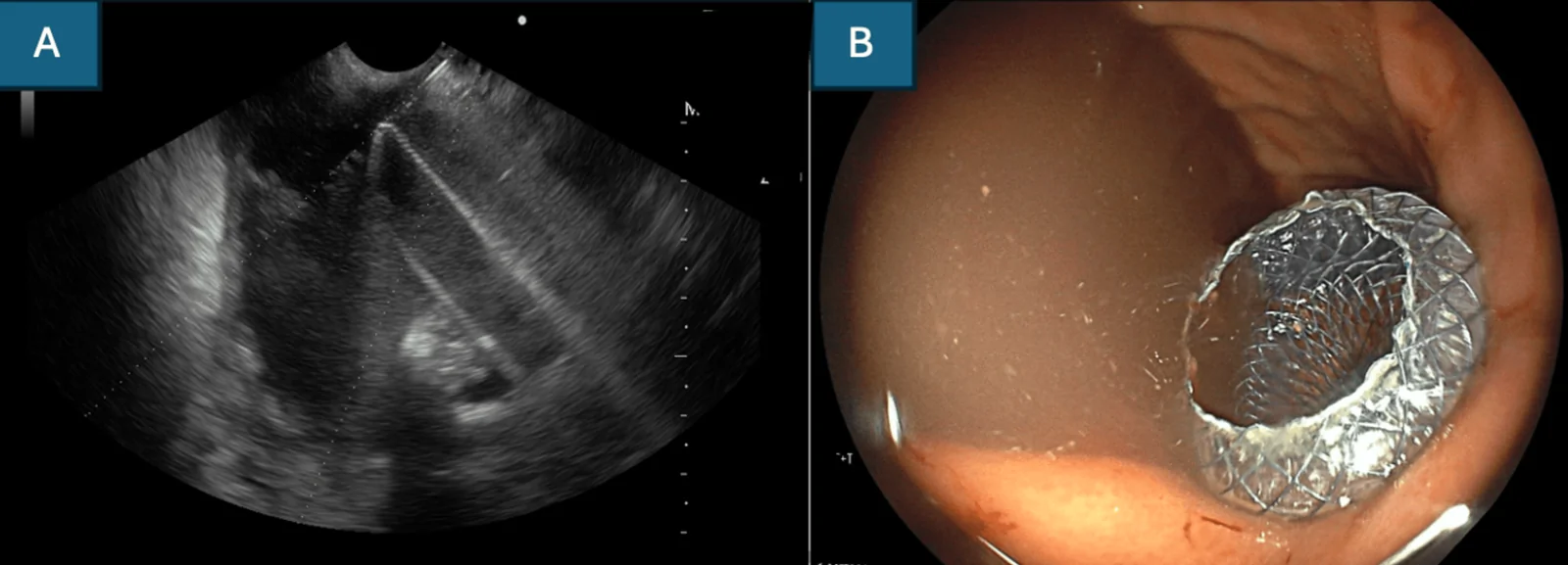
Walled-off necrosis (WON). (A) Endoscopic ultrasound (EUS) view with the stent in situ. (B) Necrotic content drained into the gastric cavity.

Regarding the distribution by type of collection, pancreatic pseudocyst was the most frequent, representing 65.7% (n = 23) of cases, whereas WON accounted for 34.3% (n = 12) (Figures 1, 2).

Among patients with pancreatic pseudocysts, 13.0% (n = 3) presented with infection at the time of drainage, evidenced by the presence of purulent material and debris, without evidence of necrotic tissue.

Regarding stent type, the most commonly used was the double pigtail plastic stent, employed in 60.0% (n = 21) of cases, while lumen-apposing metal stents (LAMS) were used in 40.0% (n = 14). This distribution was mainly related to device availability at the time of the procedure rather than to differences in efficacy between stent types (Table 1).

**Table 1:** Distribution by type of fluid collection and technical aspects. WON, walled-off necrosis; LAMS: lumen-apposing metal stent; N, number of cases; %, percentage equivalent.

Variable	n (%)
Collection type	
Pseudocyst	23 (65.7%)
WON	12 (34.3%)
Pseudocyst infection	
Yes	3 (13.0%)
No	20 (87.0)
Necrosectomy	
Yes	3 (8.6%)
No	32 (91.4%)
Stent type	
Double plastic pigtail	21 (60.0%)
LAMS	14 (40.0%)
Transmural drainage	
Stomach	34 (97.1%)
Duodenum	1 (2.9%)

The mean stent indwelling time was 35.31 ± 9.58 days, with a minimum of 27 days and a maximum of 61 days. The mean post-procedural hospital stay was 3.5 days. Direct endoscopic necrosectomy was performed in 8.6% of cases (n = 3), corresponding to patients who did not show adequate clinical improvement after the initial transmural drainage.

**Figure 3:**
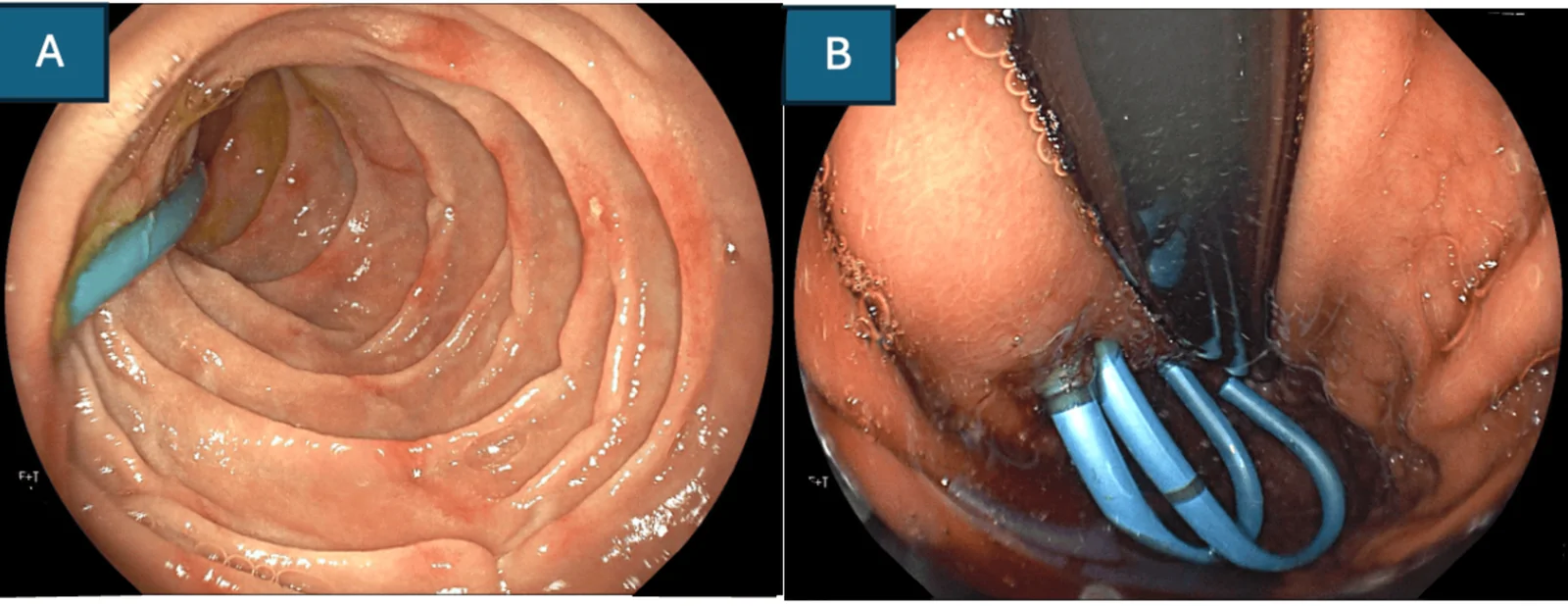
Use of a double-pigtail plastic stent. (A) Transmural drainage into the duodenum. (B) Transmural gastric drainage.

From a technical standpoint, transmural drainage was predominantly performed via the stomach in 97.1% of cases (n = 34), whereas only one case (2.9%) was performed via the duodenum (Table 1, Figure 3).

Technical success of endoscopic management with stent placement was achieved in 100% of cases (n = 35), while recurrence of peripancreatic collections occurred in three patients, representing 8.6% (n = 3) of the cohort.

Complications were reported in only 5.8% of patients (n = 2), corresponding to one case of bleeding and one case of stent obstruction, each accounting for 2.9%. Both complications were successfully managed: bleeding with hemostasis using hemoclips, and stent obstruction through stent removal and lumen cleaning.

No cases of technical failure were recorded (0.0%), and there was no 30-day mortality (n = 0).

## Discussion

Regarding the type of collection, a higher proportion of pancreatic pseudocysts was observed, which is similar to the report by Varadarajulu et al., who found a predominance of pseudocysts (45%) [13]. This finding is expected, as pseudocysts are a frequent complication of acute pancreatitis and have also been reported in up to 25% of cases of chronic pancreatitis [14].

However, it should be noted that not all pancreatic pseudocysts require drainage. According to the guidelines of the International Association of Pancreatology/American Pancreatic Association, the American Gastroenterological Association, and the American Society for Gastrointestinal Endoscopy, intervention is recommended only in symptomatic pseudocysts and infected collections [14-17]. Although these recommendations have evolved, early drainage is now allowed in selected cases (before four weeks), such as large symptomatic collections causing pain or obstruction, or those with an active infectious focus refractory to medical therapy.

Among the interventions performed, a necrosectomy rate of 8.6% was observed. This low prevalence can be explained by the fact that, unlike pseudocysts, necrotic collections are more viscous and contain large amounts of debris, making drainage more difficult and requiring a more complex endoscopic approach [18].

In our study, the most commonly used stent was the double pigtail plastic stent, and a LAMS was used in only 40% of cases. This distribution was mainly related to device availability at the time of the procedure rather than superiority of one device over another. However, in accordance with the literature, there is no single “best” stent; rather, each device has specific advantages depending on the type of collection, the presence of necrosis, and anatomical considerations. One advantage of LAMS is that it provides easy access for endoscopic debridement without the need for exchange [19]. 

Some authors have described a combined approach consisting of initial placement of a LAMS, allowing drainage of dense material, followed by removal after three to four weeks once the cavity has decreased in size, in order to reduce the risk of bleeding. This is subsequently replaced by one or two plastic pigtail stents to maintain tract patency for a longer period until complete drainage and closure of the cavity are achieved [20].

This approach was used only in selected cases of incidental LAMS removal during necrosectomy. Furthermore, rather than the type of stent used, the primary goal was to maintain a patent fistulous tract for a sufficient period to allow repeated necrosectomies when necessary and to ensure adequate resolution of the collection.

Transmural drainage was most frequently performed via the stomach (97.1%), showing no variability compared with current literature and in agreement with reports by Varadarajulu et al., where most drainages were transgastric (81.6%), followed by transduodenal (13.2%), transesophageal (4.2%), and transjejunal (1%), as well as the study by Tilara et al., in which transgastric drainage was performed in 97% of patients and transduodenal drainage in the remaining 3% [13,20]. Nevertheless, the approach may vary depending on technical feasibility, as well as the size and location of the collection.

Regarding technical success, it was achieved in 100% of cases, demonstrating the feasibility of stent placement from the initial endoscopic procedure. This finding is consistent with the clinical literature, where endoscopic intervention success rates have been reported between 85% and 93% [13-21]. This high success rate is comparable to other reports and reinforces that EUS-guided techniques are highly effective for the drainage of peripancreatic collections.

The mean post-procedural hospital stay was 3.5 days; however, this outcome was influenced by factors inherent to hospital care and not directly related to the endoscopic procedure itself.

Recurrence was observed in three cases (8.6%), which falls within the range reported in the literature (5%-32%) [13-21]. It has been described that a significant proportion of recurrences are related to unresolved underlying pancreatic duct pathology, namely ductal leakage (disconnected pancreatic duct syndrome) or pancreatic duct stenosis, making it an important indicator of procedural effectiveness.

The complication rate was 5.8%, which is lower than that reported in the literature, where complication rates associated with endoscopic procedures range from 6% to 11% [13-21]. One case of bleeding and one case of stent obstruction were reported. This finding is partially consistent with the literature, where the most common complications include hemorrhage and infectious events, as described by several authors, including Hookey et al., who reported bleeding (n = 6), pneumoperitoneum (n = 4), systemic infection, duodenal communication requiring surgical drainage, and post-ERCP pancreatitis (n = 1 each) [21].

Similarly, Arvanitakis et al. reported transient bleeding requiring transfusion and retroperitoneal leakage without clinical impact (n = 1 each) [22].

Finally, no mortality was reported in this study; however, isolated cases have been described in the literature, such as in the study by Hookey et al., where a 5% mortality rate was observed [21]. Therefore, this series demonstrated a lower mortality rate compared with some previously published reports.

Regarding the strengths of the study, the three-year study period allowed for the inclusion of a representative patient population, mainly due to the tertiary care and referral center status of the institution. This setting ensures an adequate volume of patients with more complex pathology, thereby contributing to greater experience in these types of endoscopic approaches.

Although the main limitation of this study is its retrospective design, which implies methodological constraints in the standardization of assessments, it can be concluded that there is adequate experience in the management of peripancreatic collections at this institution, with findings and outcomes comparable to those reported in the international literature.

## Conclusions

The findings of this study demonstrate the efficacy and safety of endoscopic intervention in the management of peripancreatic collections, highlighting that endoscopic drainage as a first-line option in patients with this condition is highly feasible. However, the most relevant contribution of our study is the use of endoscopic ultrasound (EUS), which has not only become the standard of care but is also currently considered indispensable, as drainage without EUS guidance is no longer deemed feasible due to its superior ability to localize and characterize the lesion. This has rendered blind puncture approaches obsolete, less feasible, and associated with higher risk.
